# Exploring the Constituent Elements of a Successful Mobile Health Intervention for Prediabetic Patients in King Saud University Medical City Hospitals in Saudi Arabia: Cross-sectional Study

**DOI:** 10.2196/22968

**Published:** 2021-07-20

**Authors:** Fayz Alshehri, Fahdah Alshaikh

**Affiliations:** 1 Executive Department of Information Technology King Saud University Medical City King Saud University Riyadh Saudi Arabia; 2 Community Health Department, Applied Medical Sciences King Saud University Riyadh Saudi Arabia

**Keywords:** prediabetes, mHealth, CeHRes roadmap, Saudi Arabia

## Abstract

**Background:**

Self-management of prediabetic patients is crucial since they are at high risk of developing type 2 diabetes. Mobile health (mHealth) apps could contribute to potentially reducing the burden of diabetes by supporting the self-management of prediabetic patients.

**Objective:**

This study aimed to explore the constituent elements of a successful mHealth intervention for prediabetic patients in King Saud University Medical City (KSUMC) hospitals in Saudi Arabia using the Centre for eHealth Research (CeHRes) roadmap.

**Methods:**

This study used the CeHRes roadmap as a developmental guideline for proposing mHealth app features for self-management of prediabetic patients and was performed in 3 phases with one round in each phase. First, a contextual inquiry was conducted via an online self-administered questionnaire for both health care providers and patients. Second, the value specification phase elaborated on the outcomes from the contextual inquiry phase. Finally, prototype user design was performed in cocreation with end users. The design phase was also conducted via an online self-administered questionnaire to evaluate the proposed features of mHealth apps by prediabetic patients.

**Results:**

A total of 20 health care providers participated in the study. The results revealed that the most powerful intervention for prediabetes was a combination of medication, physical activity, and healthy diet plans (12/20, 60%). Furthermore, the most common challenge faced by prediabetes patients was patient adherence to healthy diet and physical activity recommendations (10/20, 50%). Almost all patients believed that mHealth apps would be useful for prediabetic patients. A total of 48 prediabetic patients participated in the study. The results indicated that the most powerful intervention for prediabetic patients is a combination of healthy diet and physical activity plans (21/48, 44%), and the most frequent challenge that may lead the patients to discontinue the current intervention was the commitment to a physical activity plan (35/48, 75%). Furthermore, 15% (17/48) of patients use well-being and health apps to manage their current health status. The most common difficulties faced by the patients were navigating app features (mean 2.02 [SD 1.7]) followed by the app language (mean 1.88 [SD 2.0]); these difficulties occurred at a significantly higher rate among those with secondary or lower educational levels as compared to undergraduate and postgraduate levels (*P*<.05). Finally, the features proposed in the prototype design scored more than 2.5 points higher and indicate the need for these features to be included in the mHealth app.

**Conclusions:**

This study aimed to provide real-world insights into the development of an mHealth app for a diabetes prevention intervention by involving both health care providers and prediabetic patients in KSUMC hospitals. Therefore, the proposed app, which comprises all necessary features, may aid patients with prediabetes in self-management and making changes in their lifestyle.

## Introduction

### Background

Diabetes mellitus (DM) is one of the fastest growing health problems worldwide, and it has been associated with adverse health outcomes such as rising obesity, reduced physical activity, and mortality [1,2]. According to the International Diabetes Federation, the number of adults (aged 20 to 79 years) with diabetes worldwide was 463 million in 2019; by 2045, this number will increase to 700 million [3,4]. Furthermore, the worldwide prevalence of underdiagnosed diabetes (prediabetes) was estimated as 50.1% in 2019 [3,4]. Compared to overseas countries, Saudi Arabia has a high prevalence of DM, and the World Health Organization has ranked it as the seventh highest country for the prevalence of DM [5]. Approximately 4.3 million adults have diabetes in 2019; by 2045, this will increase to about 7.9 million, and the estimated age-adjusted comparative prevalence of DM was 15.8% in 2019, which will rise to 17.8% by 2045 as reported by the International Diabetes Federation [4]. In the meantime, the number of patients with prediabetes was approximately 1.7 million in 2019 in Saudi Arabia with a prevalence of 39% [4]. Prediabetes refers to a person who a higher than normal blood sugar level that is not high enough to be considered diabetic yet. However, without lifestyle changes, individuals with prediabetes are more likely to develop the disease. This indicates that prediabetic patients are at relatively high risk for developing diabetes in the future [6].

It is believed that self-management of prediabetes might play a vital role in preventing or delaying the development of the disease and its adverse effects. Components of self-management include diabetes education, healthy eating, physical activity, medication, and device use [7]. For instance, any increase from a low level of physical activity can reduce the incidence of developing diabetes [8]. One popular lifestyle change program directed toward prediabetic patients is the one modeled after the Diabetes Prevention Program research study [9,10].

Recent years have seen a growing trend in the availability and use of well-being and health apps. Mobile health (mHealth), “the use of mobile communications for health information and services” [11], can play a significant role in adjusting and improving health promotion lifestyle, prevention of disease, and disease self-management [12-16]. That is, mHealth is characterized by the mobile technology’s mobility, which facilitates instantaneous access and direct communication allowing for faster transfer of health information, which in turn supports medical and public health practices [17]. In the diabetes context, mHealth is a promising technology that supports patient engagement in their health care since most people own and regularly use a mobile phone and may use functions like text and voice messaging with health care professionals, connections to external devices (eg, heart rate measurement and monitoring of blood glucose or blood pressure), medication support, tracking physical activity (eg, lifestyle tracking using pedometer technologies to track one’s steps and calories), and monitoring healthy diet behavior via mHealth apps [18-22]. A systematic review assessed the efficacy, usability, and features of commercially available mHealth apps for self-management of diabetes and showed that none of the reviewed studies exhibited significant patient improvements in quality of life, BMI, and blood pressure.

There is extensive literature on the adoption of mHealth apps to illustrate influences on the adoption decision [23-35]. Some studies were based on the technology acceptance model [23-29]. Other studies relied on the unified theory of acceptance and use of technology [30-32]. Little research took into account the appropriation and implementation perspective on the use of mHealth apps; these studies mostly focusing on continued use and failed to consider the integration of multifaceted everyday life patterns [28,33,34]. A study by Rossmann et al [35] used the mobile appropriation model to investigate how diabetes patients use mHealth apps for self-management and showed that patients are heterogeneous in evaluating such mHealth apps. Some studies relied on the Centre for eHealth Research (CeHRes) roadmap [22,23]. According to the framework by Klasnja and Pratt [22], there are 5 behavioral intervention strategies enabled by smartphones. These include tracking health information (eg, setting goals for targeted behavior, monitoring, tracking, reminders, and progress visualization), involving the health care team (eg, sharing health information with health care providers), leveraging social influence (eg, social networking), increasing the accessibility of health information (eg, access to didactic curriculum, coaching), and using entertainment (eg, reward-based games). Thus, most existing frameworks were found to rely more on a conceptual approach instead of practical guidelines and lack of participatory approach that ensure the eHealth technologies are stakeholder-driven [23]. Furthermore, studies were largely heterogeneous and had to some extent methodological issues including inconsistency in randomization reporting, masking, and allocation and low quality that hurt interpretation of the results [20,21,23]. Therefore, to avoid failure of making an impact with these eHealth technologies, eHealth developers and researchers should adopt a reliable approach in the early stage of development. The behavioral change intervention strategy was also used and showed it was an effective tool for developing mHealth apps [36,37].

The preliminary research in the literature shows that there is a lack of evidence on the use of mHealth technologies in the prevention of type 2 diabetes in Saudi Arabia [38]. Therefore, this study aims to provide an initial step toward building an mHealth technology that could support the current prediabetes self-management intervention. More specifically, this study is devoted to determining the requirements and specifications needed to design an mHealth intervention for prediabetic patients in King Saud University Medical City hospitals. This study should contribute to the overall vision of Saudi Arabia’s Ministry of Health in reducing the burden of type 2 diabetes [39].

### Centre for eHealth Research Roadmap

Van Gemert-Pijnen et al [23] proposed a holistic framework for the development of eHealth technologies which are the outcomes of a systematic review of existing frameworks and from empirical research and progressive insights about the framework obtained from experts at eHealth conferences. The framework is also referred to as the CeHRes roadmap. It serves as an evidence-based roadmap to the research and developmental activities involved in developing eHealth technologies from concept definition through development to summative evaluation [40]. This roadmap aims to ensure the developed eHealth technologies are human-centered, tailored to stakeholder needs, and capable of altering user behaviors, thus increasing the uptake and impact of eHealth technologies. The CeHRes roadmap takes an iterative approach through 5 phases of development: contextual inquiry, value specification, design, operationalization, and summative evaluation to ensure an iterative, flexible, and dynamic process resulting in concepts of the technology (from ideation to product) [23].

Identifying user values is one key task in developing eHealth technologies, which are a key aspect of the CeHRes roadmap. Despite well-established literature on consumer needs, it is often not clear what needs to be addressed [41]. Studies that used the CeHRes roadmap reported that they benefit from the iterative approach provided by the framework which allows them to use it as a checklist tool afterward [42]. This also allows creating outcome benchmarks meaning that if the design requirement does not meet the targeted goals of the eHealth technology, one can return to different points within the CeHRes phases to make adjustments [41].

By 2017, the use of the CeHRes roadmap in the development of eHealth technologies was apparent in 26 studies but also recognized and referenced in hundreds of studies. The app of the CeHRes roadmap was mostly for the control of infectious diseases, management of cancer, treatment of mental health, and management of diabetes [43].

## Methods

### Overview

**Figure 1:**
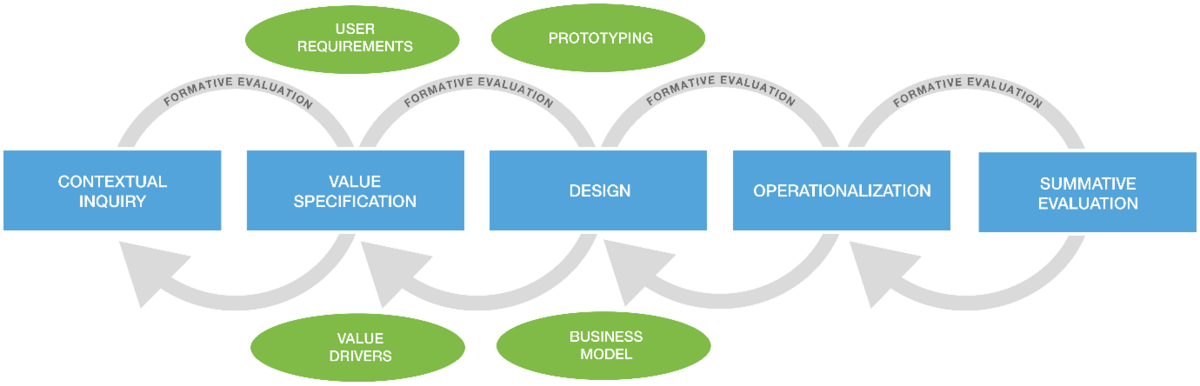
The CeHRes roadmap.

This is a cross-sectional study using self-administered questionnaires as the main tools for collecting data and the CeHRes roadmap as a guide. For this study, the first 3 phases were conducted: contextual inquiry, value specification, and design phases. According to the CeHRes roadmap, the operationalization and summative evaluation phases are concerned with the introduction, adoption, and employment of the technology in practice and evaluating how it is being used and its effects. Therefore, they are beyond the scope of this study, which is limited to designing a low fidelity prototype that reflects the requirements and specifications for the mHealth intervention for prediabetic patients in KSUMC hospitals (Figure 1).

### Methodological Framework

The first phase of the CeHRes roadmap is the contextual inquiry. This phase aims to understand the current issues, how the technology can contribute to resolving these issues, and who might benefit from the technology. This can be realized by investigating 3 subphases: stakeholder identification, stakeholder analysis, and describing the current situation [44]. In this study, we initially identified and analyzed key stakeholders such as physicians, dietitians, health educators, and prediabetic patients before we explored the current situation for the prediabetes intervention in KSUMC hospitals. The current situation then was explored using self-administrated questionnaires sent to health care providers and prediabetic patients.

The second phase of the CeHRes roadmap is value specification which elaborates on the outcomes from the contextual inquiry. The value specification has two main outcomes: a value map that contains the values that mHealth should address and a list of requirements [45]. These requirements are needed to develop the technology, which is in our case is the mHealth app for a prediabetes intervention. In this phase, the key stakeholder values and requirements were realized and ranked based on the importance and need using descriptive statistics. The findings helped us to understand the added value that the proposed mHealth features would provide for prediabetic patients.

The outcomes of the contextual inquiry and value specification phases were then translated into a blueprint for our proposed mHealth technology, which was developed in the design phase. According to the CeHRes roadmap, the design of any eHealth technology should consist of 3 subphases: develop low-fidelity and high-fidelity prototypes, conduct usability tests, and add persuasive elements [46]. For this study, a low-fidelity prototype was developed that addressed the values and requirements from the previous phases. Table 1 describes how the proposed mHealth features were guided by Klansja and Pratt’s framework of 5 behavioral intervention strategies enabled by smartphones as well as the key components of a lifestyle change program in preventing type 2 diabetes [9,22]. These proposed mHealth features were then translated into a prototype of user interfaces. The prototype was then tested by 2 mobile app developers on the design level.

**Table 1:** Proposed mHealth features guided by lifestyle change plan of Kirley & Sachdev and Klansja and Pratt’s framework.

Kirley & Sachdev lifestyle change plan to prevent diabetes	Klansj and Pratt’s framework	Proposed mHealth features
Self-monitoring of diet and physical activity	Tracking health information	Weight management Step counting Blood glucose calculator List of healthy food Notifications
Didactic curriculum and coaching	Involving health team	Phone call Text messaging
Peer support	Leveraging social media	Peer and group communication
Individual skills development and problem solving	Accessibility of health information	Pop-up quizzes Frequently asked questions
Motivation	Using entertainment	Users collecting points for the following features Weight measurement (reaching goal) Step counting (reaching goal) Pop-up quizzes (questions answered)

The end users for our proposed mHealth app are prediabetic patients. Therefore, the prototype user interfaces were sent to patients using an online form to evaluate them one by one on a 5-point Likert-type scale from “I strongly need it” to “I do not need it at all.” The design of the prototype has considered the need for persuasive elements in the proposed mHealth features, which are introduced as motivation features such as collecting points and getting badges when a certain goal was achieved.

### Participants

The populations of this study comprised health care providers from both KSUMC hospitals and prediabetic patients. The health care providers were eligible if they are involved in the management of prediabetic patients. A total of 40 health care providers working in the primary care departments in the KSUMC hospitals were eligible: physicians (28/40), dietitians (8/40), health educators (4/40). The prediabetic patient population inclusive criteria were patients diagnosed with prediabetes, Arabic speakers, age group from 20 to 65 years, and receiving intervention for prediabetes. The age range used is the frequent age of onset of diabetes [1], and 1040 prediabetic patients were found eligible for this study in the hospitals’ electronic health records.

The sample size for health care providers population was 38 and for prediabetic patients it was 281. A convenience sampling technique was used for health care providers and simple random sampling was performed on the prediabetic patients list.

### Data Collection Tools

Two self-administrated questionnaires were used in this study; one for health care providers and one for prediabetic patients. The first questionnaire was sent to health care providers in paper-based form to explore the current situation from the health care provider side. The second questionnaire was an online questionnaire that was sent by text message to patient phones, and it aimed to explore the current situation from the prediabetic patient side. Both questionnaires were developed using the information gathered during the stakeholder identification and analysis subphases in the contextual inquiry phase. This information was collected by interviewing 3 health care providers, a physician, dietitian, and health educator from the primary care departments in KSUMC hospitals. For the prediabetic patient questionnaire, 3 prediabetic patients were interviewed (male aged 58 years, male aged 32 years, and female aged 31 years). All questionnaires were then piloted for clarity of questions (Multimedia Appendix 1 and 2).

Moreover, the prototype of user interfaces was also reviewed by the same group of patients for clarity of the presentation of user interfaces of the proposed mHealth features (Multimedia Appendix 3). The instruments were sent to participants for a duration of 1 week and with a reminder 2 days before the closing time of the online form.

### Data Analysis

The data were analyzed using SPSS (version 25, IBM Corp) software. Descriptive statistics were presented as means and standard deviations for continuous variables and as frequencies and percentages for categorical variables. Independent sample *t* tests and 1-way analysis of variance tests were performed to test for differences in mean scores as appropriate. Statistical significance was sought at values lower than 5%.

### Ethical Consideration

This study has received the ethical approval number E-19-4118 from the Institute of Review Board at College of Medicine in King Saud University (Multimedia Appendix 4).

## Results

### Health Care Provider Questionnaire

A total of 20 questionnaires were completed and received: 10 physicians (50%), 6 dietitians (30%), and 4 health educators (20%). The majority of respondents were females (16/20, 80%) and aged 30 to 39 years (8/20, 40%). One-half of them worked at King Khalid University Hospital (KKUH) (10/20, 50%). Most respondents stated that the most impactful intervention technique for prediabetes was a combination of medication with both healthy diet and physical exercise plans. About 65% (13/20) of participants had communicated with patients in terms of phone call (8/20, 62%), text messages (4/20, 31%), and emails (1/20, 8%). The majority of participants believed that mHealth apps would help prediabetes patients (17/20, 85%; Table 2).

Furthermore, health care providers were asked about the challenges and barriers using open questions. The health care provider answers were then analyzed using quantitative text analysis to categorize the barriers and challenges facing the current interventions for prediabetic patients in KSUMC hospitals. The results showed 5 common reported barriers and challenges to the current intervention. The lack of adherence to a healthy diet and physical activity plans was the most common barrier (10/20, 50%). This is followed by the lack of awareness (8/20, 40%). Accordingly, lack of awareness was described frequently as the patients denying the fact that they had a high blood sugar level or not taking the medical diagnosis seriously. Furthermore, loss of follow-up (7/20, 35%) and lack of motivation (5/20, 25%) were found among the barriers faced by patients. Finally, the participants also reported that there were cultural barriers facing patients to adhere to medical recommendations (4/20, 20%; Table 2).

**Table 2:** Descriptive statistics of health care providers questionnaire (n-20).

Characteristics		Value, n (%)
**Occupation**		
	Dietitian	10 (50)
	Physician	6 (30)
	Health educator	4 (20)
**Gender**		
	Male	6 (30)
	Female	14 (70)
**Age group (years)**		
	20-29	4 (20)
	30-39	6 (30)
	40-49	8 (40)
	50-59	2 (10)
**Hospital**		
	King Khalid University Hospital	10 (50)
	King Abdulaziz University Hospital	10 (50)
**What are the most impactful intervention techniques you used in your clinic for patients with prediabetes?**		
	Medication and healthy diet plan	1 (5)
	Medication, healthy diet plan, and physical exercise plan	12 (60)
	Healthy diet plan and physical exercise plan	7 (35)
**Do you or any of your team members communicate with patients remotely? If so, by what means?**		
	No	7 (35)
	Yes	13 (65)
	Phone call	8 (62)
	Text messages	4 (31)
	Emails	1 (8)
**Health care provider perceptions of mobile health technologies**		
	No, I do not believe it can help them	0 (0)
	Not sure if it can help them	3 (15)
	Yes, I believe it can help them	17 (85)
**Barriers or challenges facing the current interventions for prediabetic patients to prevent diabetes**		
	Adherence to healthy diet and physical activity plans	10 (50)
	Lack of awareness	8 (40)
	Loss of follow-up	7 (35)
	Lack of motivation	5 (25)
	Cultural barriers	4 (20)

### Prediabetic Patient Questionnaire

A total of 48 participants responded to the questionnaire (Table 3). Two-thirds of participants were males (32/48, 67%). One-half of them were aged 50 years or above (24/48) and 46% (22/48) had undergraduate educational level. One-third (16/48) of respondents were diagnosed with prediabetes more than five years ago. The majority (37/48, 77%) of patients who participated in this questionnaire were from KKUH.

The majority (21/48, 44%) of participants were engaged with lifestyle change programs including a healthy diet plan and physical activity plan as an intervention strategy for diabetes. Meanwhile, the less frequent intervention strategy was the combined one of medication, healthy diet, and physical activity plans (7/48, 15%).

The majority (35/48, 75%) of participants reported that the most frequent challenges and barriers leading prediabetic patients to discontinue the current intervention were the commitment to physical activity plan. This is followed by commitment to healthy dietary plan (25/48, 52%), commitment to constantly following up in clinic (12/48, 25%), and lack of self-motivation (11/48, 23%). However, the less frequent challenge and the barrier was the commitment to take medication on time (7/48, 15%).

The results also revealed that most (31/48, 65%) respondents stated that they did not use mHealth apps to manage their status of health. On the other hand, 15% (17/48) of respondents stated they use mHealth apps either usually or sometimes to manage their current status of health, and some of them mentioned some apps including iHealth, VitaDock, Fitbit, Nike Run, Samsung Health, and Apple Health and others mentioned they use apps for Zumba training for fitness and weight loss. Most participants reported that they used websites using search engines (30/48, 63%) while social media platforms such as Facebook, Twitter (20/48, 42%), and YouTube (23/48, 48%) had been used sometimes. The majority (36/48, 75%) of respondents exhibited their willingness and readiness to use mHealth apps while 25% (12/48) of them stated that they may use these apps, but none of them stated that they will not use such apps (Table 3).

Moreover, the results indicate that the most downside and difficulty of using such mHealth apps was navigating the features in mHealth apps whereby it scored a mean of 2.02 (SD 1.7) points. The mean score of app language difficulty was 1.88 (SD 2.0) points, 1.81 (SD 1.6) points for self-motivation to use the mHealth apps, 1.77 (SD 1.7) points for understanding the goal of mHealth apps, and 1.71 (SD 1.7) points for learning how to use these apps (Table 4). Nevertheless, these average scores were less than the average score of 2.5 points (ie, 15/6) indicating that these difficulties were mild. The results also revealed that there were no statistically significant differences in average scores of these difficulties by gender, age, and educational levels. Males were more likely to have difficulty in self-motivation than female counterparts (*P<.*05). However, the only differences found to be statistically significant were self-motivation by gender (*P*<.05) and app language by educational levels (*P*<.05). That is, the average score of self-motivation of males was higher than that for female counterparts. Moreover, the average score of language difficulty for participants with secondary or lower education was higher than those with undergraduate and postgraduate educational levels (Table 5).

**Table 3:** Descriptive statistics of the sample (n=48).

Item characteristics				Value, n (%)
**Gender**				
	Male		32 (67)	
	Female		16 (33)	
**Age group (years)**				
	20-29		2 (4)	
	30-39		9 (19)	
	40-49		13 (27)	
	50 and above		24 (50)	
**Educational levels**				
	Secondary or lower		12 (25)	
	Undergraduate		22 (46)	
	Postgraduate		14 (29)	
**History of diagnosis**				
	Less than 1 year		11 (23)	
	1-2 years		9 (19)	
	2-3 year		9 (19)	
	3-5 years		3 (6)	
	More than 5 years		16 (33)	
**Hospital**				
	King Khalid University Hospital		37 (77)	
	King Abdulaziz University Hospital		11 (23)	
**Intervention strategy for prediabetes**				
	Only medication		10 (21)	
	Medication + healthy diet plan		10 (21)	
	Medication + healthy diet plan + physical activity plan		7 (15)	
	Healthy diet plan + physical activity plan		21 (44)	
**Challenges and barriers**				
	Commitment to physical activity plan		35 (73)	
	Commitment to a healthy dietary plan		25 (52)	
	Commitment to constantly following up in the clinic		12 (25)	
	Lack of self-motivation		11 (23)	
	Commitment to take medication on time		7 (15)	
**Frequent use of mHealth Apps**				
	Always		7 (15)	
	Sometimes		10 (21)	
	No		31 (65)	
**Frequent use of platforms**				
	**Websites using the search engine**			
		Always	30 (63)	
		Sometimes	12 (25)	
		Never	6 (13)	
	**Social media (ie, Twitter, Facebook, WhatsApp, telegram)**			
		Always	14 (29)	
		Sometimes	20 (42)	
		Never	14 (29)	
	**YouTube**			
		Always	11 (23)	
		Sometimes	23 (48)	
		Never	14 (29)	
**Readiness to use mHealth app among prediabetic patients**				
	Yes		36 (75)	
	No		0 (0)	
	Maybe		12 (25)	

**Table 4:** Downside and difficulty faced by participants while using mHealth apps.

Type of difficulties	Mean (SD)
App language (eg, app does not support the Arabic language)	1.88 (2.0)
Learning how to use the mHealth app	1.71 (1.6)
Understand the goal of the mHealth app	1.77 (1.7)
Navigating the features in the mHealth app	2.02 (1.7)
Self-motivation to use mHealth app	1.81 (1.6)

**Table 5:** Mean scores of each difficulty by gender, age groups, and educational levels.

Type of difficulties			App language		App learning		App understanding		App navigation		Self-motivation
**Gender**											
	Male	2.09 (1.9)		2.0 (1.6)		2.09 (1.7)		2.25 (1.6)		2.16 (1.6)^a^	
	Female	1.44 (2.4)		1.13 (1.4)		1.13 (1.5)		1.56 (1.7)		1.13 (1.2)	
**Age groups**											
	20-29	0.0 (0)		2.0 (1.4)		1.50 (0.7)		3.50 (2.1)		2.50 (2.1)	
	30-39	3.22 (2.0)		1.89 (1.4)		2.11 (1.6)		2.22 (1.5)		2.56 (1.3)	
	40-49	1.92 (1.9)		1.69 (1.7)		1.62 (1.8)		1.62 (1.3)		1.38 (1.6)	
	50 years and above	1.5 (1.9)		1.63 (1.7)		1.75 (1.8)		2.04 (1.9)		1.71 (1.6)	
**Educational levels**											
	Secondary or lower	3.33^a^ (1.9)		2.42 (1.7)		2.33 (1.7)		2.08 (1.7)		1.83 (1.4)	
	Undergraduate	1.41 (1.7)		1.32 (1.4)		1.32 (1.6)		1.82 (1.9)		1.59 (1.8)	
	Postgraduate	1.36 (1.8)		1.71 (1.7)		2.00 (1.8)		2.29 (1.4)		2.14 (1.4)	

^a^Significant at 5% level of significance.

### Prediabetic Patient Evaluation of the Prototype User Interfaces

Table 6 presents the average score given for each feature along with their corresponding lifestyle change program components and the 5 smartphone behavioral intervention strategies by Klansja and Pratt as well as the key components of lifestyle change program in preventing type 2 diabetes [9,22]. The findings indicate that the mean score of tracking health information items (ie, self-monitoring of diet, physical activity, and weight component) ranged from 3.88 (SD 0.7) to 4.54 (SD 1.4) points. Furthermore, respondents scored average scores of 4.15 (SD 0.9) and 4.37 (SD 0.9) for involving health teams in terms of the phone call and text messages features, respectively. The mean scores of accessibility of health information (ie, individual skills development and problem solving) were 4.15 (SD 1.0) for pop-up quizzes and 4.37 (SD 0.8) for frequently asked questions about the prevention of diabetes. The entertainment utility (ie, motivation, collecting points, and getting badges when a certain goal was achieved in such features) had mean scores ranged from 3.92 to 4.15 (SD 1). That is, features like getting points and badges when completing daily steps and walking time needed weekly received an average score of 4.15 (SD 1.0) points, getting points and badges when achieving 5% loss of body weight had a mean score of 4.13 (SD 1.0) points, and getting points and badges by answering the pop-up quizzes correctly had an average score of 3.92 (SD 1.0) points. However, the social communication features had the lowest scores reported by respondents for both peer support (mean 3.58 [SD 1.0]) and group support (mean 3.62 [SD 1.0]). Generally speaking, the prediabetic patients response scores of these features were moderate and indicate the need for these features to be available in mHealth apps.

**Table 6:** Average scores of each feature along with their correspondents lifestyle change program components and the 5 smartphone behavioral intervention strategies by Klansja and Pratt [9,22].

Feature and component		Value, mean (SD)
**Tracking health information (self-monitoring of diet, physical activity, and weight)**		
	Step count	4.54 (0.7)
	Blood glucose calculator	3.88 (1.4)
	List of daily healthy diet options	4.54 (0.7)
	Notifications	4.37 (0.9)
	Weight and body mass management	4.46 (0.9)
**Involving health team**		
	Phone call communication	4.15 (0.9)
	Text messages communication	4.37 (0.9)
**Accessibility of health information (individual skills development and problem solving)**		
	Pop-up quizzes	4.15 (1.0)
	Frequent asked questions about the prevention of diabetes	4.37 (0.8)
**Using entertainment (motivation; collecting points and getting badges when a certain goal was achieved in the following features)**		
	Weight and body mass management (when achieving 5% body weight loss)	4.13 (1.0)
	Step count (completing daily steps & walking time needed weekly)	4.15 (1.0)
	Pop-up quizzes (when correctly answering the quizzes)	3.92 (1.0)
**Leveraging social media**		
	Group support	3.58 (1.0)
	Peer support	3.62 (1.0)

The results also indicate that there were no statistically significant differences in mean scores of these proposed features by gender, age groups, and educational levels (*P<.*05) (Tables 7, 8, and 9). That is, participants have the same perceptions toward these features regardless of their gender, age, and educational levels.

**Table 7:** Average scores of each feature by gender.

Feature and component		Male, mean (SD)	Female, mean (SD)	*P* value
**Tracking health information (self-monitoring of diet, physical activity, and weight)**				
	Step count	4.55 (0.6)	4.53 (0.8)	.95
	Blood glucose calculator	3.59 (1.4)	4.10 (1.3)	.18
	List of daily healthy diet options	4.50 (0.6)	4.57 (0.7)	.73
	Notifications	4.27 (0.9)	4.23 (0.9)	.54
	Weight and body mass management	4.64 (0.7)	4.33 (1.0)	.24
**Involving health team**				
	Phone call communication	4.18 (1.0)	4.13 (0.9)	.06
	Text messages communication	4.41 (1.0)	4.33 (0.9)	.08
	Pop ups quizzes	4.32 (0.8)	4.03 (1.0)	.31
**Accessibility of health information (individual skills development and problem solving)**				
	Frequent asked questions about the prevention of diabetes	4.59 (0.7)	4.20 (0.9)	.10
**Using entertainment (motivation; collecting points and getting badges when certain goal was achieved in the following features)**				
	Weight and body mass management (when achieving 5% body weight loss)	4.41 (0.9)	3.93 (1.0)	.10
	Steps count (completing daily steps & walking time needed weekly)	4.27 (0.8)	4.07 (1.1)	.21
	Pop-up quizzes (when correctly answering the quizzes)	4.09 (1.0)	3.80 (1.1)	.29
**Leveraging social media**				
	Group support	3.59 (1.0)	3.57 (1.1)	.94
	Peer support	3.77 (1.0)	3.50 (1.1)	.37

**Table 8:** Average scores of each feature by age group.

Feature, component, and age group (years)			Value, mean (SD)	*P* value
**Tracking health information (Self-monitoring of diet, physical activity, and weight)**				
	**Step count**			.12
		20-29	5.00 (0.0)	—^a^
		30-39	4.83 (0.4)	—
		40-49	4.80 (0.4)	—
		50 and above	4.36 (0.8)	—
	**Blood glucose calculator**			.75
		20-29	3.67 (2.3)	—
		30-39	3.33 (1.9)	—
		40-49	4.00 (1.4)	—
		50 and above	3.97 (1.1)	—
	**List of daily healthy diet options**			.23
		20-29	4.67 (0.6)	—
		30-39	4.83 (0.4)	—
		40-49	4.80 (0.4)	—
		50 and above	4.39 (0.7)	—
	**Notifications**			.09
		20-29	5.00 (0.0)	—
		30-39	4.83 (0.4)	—
		40-49	4.70 (0.7)	—
		50 and above	4.12 (1.0)	—
	**Weight and body mass management**			.07
		20-29	5.00 (0.0)	—
		30-39	5.00 (0.0)	—
		40-49	4.80 (0.6)	—
		50 and above	4.21 (1.0)	—
**Involving health team**				
	**Phone call communication**			.78
		20-29	4.67 (0.6)	—
		30-39	4.17 (0.7)	—
		40-49	4.00 (1.2)	—
		50 and above	4.15 (0.9)	—
	**Text messages communication**			.16
		20-29	5.00 (0.0)	—
		30-39	5.00 (0.0)	—
		40-49	4.30 (0.9)	—
		50 and above	4.21 (0.9)	—
**Accessibility of health information (individual skills development and problem solving)**				
	**Pop-up quizzes**			.39
		20-29	4.00 (1.0)	—
		30-39	4.67 (0.5)	—
		40-49	4.40 (0.7)	—
		50 and above	4.00 (1.1)	—
	**Frequent asked questions about the prevention of diabetes**			.08
		20-29	5.00 (0.0)	—
		30-39	4.83 (0.4)	—
		40-49	3.90 (1.1)	—
		50 and above	4.36 (0.8)	—
**Using entertainment (motivation; collecting points and getting badges when a certain goal was achieved in the following features)**				
	**Weight and body mass management (when achieving 5% body weight loss)**			.06
		20-29	5.00 (0.0)	—
		30-39	4.83 (0.4)	—
		40-49	4.40 (0.9)	—
		50 and above	3.85 (1.0)	—
	**Step count (completing daily steps & walking time needed weekly)**			.46
		20-29	4.00 (1.0)	—
		30-39	4.50 (0.8)	—
		40-49	4.50 (0.7)	—
		50 and above	4.00 (1.1)	—
	**Pop-up quizzes (when correctly answering the quizzes)**			.53
		20-29	3.67 (1.2)	—
		30-39	4.33 (0.8)	—
		40-49	4.20 (0.9)	—
		50 and above	3.79 (1.1)	—
**Leveraging social media**				
	**Group support**			.22
		20-29	4.00 (1.0)	—
		30-39	4.33 (0.5)	—
		40-49	3.40 (1.2)	—
		50 and above	3.45 (1.0)	—
	**Peer support**			.55
		20-29	3.67 (1.2)	—
		30-39	4.17 (0.4)	—
		40-49	3.70 (1.2)	—
		50 and above	3.48 (1.1)	—

^a^Not applicable.

**Table 9:** Average scores of each feature by educational levels.

Feature, component, and education level				Value, mean (SD)		*P* value
**Tracking health information (Self-monitoring of diet, physical activity, and weight)**						
	**Step count**					.51
		Secondary or lower	4.33 (0.9)		—^a^	
		Undergraduate	4.59 (0.7)		—	
		Postgraduate	4.64 (0.5)		—	
	**Blood glucose calculator**					.21
		Secondary or lower	4.25 (1.0)		—	
		Undergraduate	3.50 (1.5)		—	
		Postgraduate	4.36 (1.1)		—	
	**List of daily healthy diet options**					.24
		Secondary or lower	4.75 (0.6)		—	
		Undergraduate	4.36 (0.7)		—	
		Postgraduate	4.64 (0.6)		—	
	**Notifications**					.68
		Secondary or lower	4.25 (1.2)		—	
		Undergraduate	4.41 (0.8)		—	
		Postgraduate	4.57 (0.9)		—	
	**Weight and body mass management**					.59
		Secondary or lower	4.50 (0.8)		—	
		Undergraduate	4.32 (1.1)		—	
		Postgraduate	4.64 (0.6)		—	
**Involving health team**						
	**Phone call communication**					.48
		Secondary or lower	4.08 (1.0)		—	
		Undergraduate	4.09 (0.9)		—	
		Postgraduate	4.43 (0.8)		—	
	**Text messages communication**					.65
		Secondary or lower	4.25 (1.1)		—	
		Undergraduate	4.45 (0.9)		—	
		Postgraduate	4.57 (0.8)		—	
**Accessibility of health information (individual skills development and problem solving)**						
	**Pop-up quizzes**					.74
		Secondary or lower	4.25 (1.1)		—	
		Undergraduate	4.00 (1.0)		—	
		Postgraduate	4.21 (0.9)		—	
	**Frequent asked questions about the prevention of diabetes**					.47
		Secondary or lower	4.17 (1.0)		—	
		Undergraduate	4.50 (0.7)		—	
		Postgraduate	4.21 (0.9)		—	
**Using entertainment (motivation; collecting points and getting badges when certain goal was achieved in the following features)**						
	**Weight and body mass management (when achieving 5% body weight loss)**					.51
		Secondary or lower	4.17 (1.1)		—	
		Undergraduate	3.95 (1.1)		—	
		Postgraduate	4.36 (0.7)		—	
	**Steps count (completing daily steps & walking time needed weekly)**					.08
		Secondary or lower	4.08 (1.4)		—	
		Undergraduate	3.86 (0.9)		—	
		Postgraduate	4.64 (0.6)		—	
	**Pop-up quizzes (when correctly answering the quizzes)**					.12
		Secondary or lower	4.08 (1.3)		—	
		Undergraduate	3.59 (0.9)		—	
		Postgraduate	4.29 (0.9)		—	
**Leveraging social media**						
	**Group support**					.38
		Secondary or lower	3.58 (1.2)		—	
		Undergraduate	3.36 (0.9)		—	
		Postgraduate	3.86 (1.0)		­—	
	**Peer support**					.07
		Secondary or lower	3.42 (1.3)		—	
		Undergraduate	3.36 (0.9)		—	
		Postgraduate	4.21 (0.9)		—	

^a^Not applicable.

## Discussion

### Principal Findings

This study is an initial development of an mHealth app for self-management of prediabetic patients in KSUMC hospitals in Saudi Arabia using a theoretically driven approach based on the CeHRes roadmap [22,23]. The main objective of this study was to determine the most important features that should be available in successful mHealth apps for self-management of prediabetes based on the CeHRes roadmap guided by the Klansja and Pratt framework and components of the lifestyle change program by Kirley and Sachdev [9,22], which is the first attempt to the best of the authors’ knowledge. Accordingly, this study builds on the perceptions of both health care providers and prediabetic patients toward the use of mHealth apps for self-management of prediabetes. From the health care provider point of view, the findings indicate that the most powerful intervention procedure for prediabetes is a combination of medication with both healthy diet and physical exercise plans. From the prediabetic patient point of view, the most powerful intervention procedure for prediabetes is a combination of both healthy diet and physical exercise plans, confirmed with the Diabetes Prevention Program goals [47]. Some studies indicated that physical activity is a key tool for the prevention and management of DM [48,49].

The majority of health care providers, as part of their management of prediabetic patients, reported that they communicated with patients via either a phone call or text messages. This is confirmed with prediabetic patients whereby most of them reported they did not use such mHealth apps while a few reported using social media platforms and websites. Communicating with patients remotely using text messaging was a common approach in delivering the curriculum of various diabetes prevention programs [47]. A recent study indicated that diabetes-related apps accounted for about 16% of the total number of available Health apps [50]. Furthermore, these diabetes-related apps differed in their functions such as tracking blood glucose measurements, physical activity, weight tracking, sharing data with clinicians or peers, social support and messaging, and nutrition database and carbohydrate tracking [51]. Veazie et al [21] indicated that even though many apps for diabetes self-management are available for commercial purposes, their study demonstrated that only 11 apps have had an impact on patient health.

In Saudi Arabia, evidence showed that the major risk factors for developing type 2 diabetes were obesity, lack of physical activity, unhealthy diet, smoking, and aging in addition to more complex factors such as lack of education, poor social support, and unhealthy environment [52]. Health education counseling by physicians was considered one of the most powerful practices in endorsing lifestyle modification such as healthy diet and physical activity as an important factor for weight management control and reduce the risk of developing diabetes [53,54]. Lifestyle modification such as increasing physical activity has the potential to not only raise glycemic control but also boost a patient’s insulin sensitivity and repair some of the damage caused by diabetes-associated complications, such as impaired cardiovascular health, one of the most common complications [55]. Lifestyle modification is also a cornerstone of any prediabetes intervention management. Evidence showed that individuals with prediabetes involved in prediabetes intervention programs have a 40% to 70% relative risk reduction of diabetes [56].

Concerning the perceived challenges and barriers of using mHealth apps in the context of prediabetes, the results revealed that the most significant challenge and barriers faced by patients reported by health care providers were the lack of adherence to a healthy diet and physical activity plans, the lack of awareness, loss of follow-up, lack of motivation, and cultural barriers facing patients to adhere to medical recommendations. On the other hand, prediabetic patients reported that the most common challenges and barriers that may lead them to discontinue the current intervention were the commitment to physical activity plan, commitment to a healthy dietary plan, commitment to constantly following up in the clinic, and lack of self-motivation. However, the commitment to take medication on time received less attention. Prediabetic patients who indicated that they have used such mHealth apps had faced difficulty in navigating the features in apps because of language barriers, self-motivation, understanding the goal, and learning how to use these apps but the levels of these difficulties were mild. The self-motivation difficulty of males was higher than their female counterparts. Moreover, prediabetic patients with lower educational levels faced app language difficulty. A recent study showed that general barriers to use of mHealth apps were evident, including financial, technical, and temporal barriers [35]. The results indicated that both health care providers and prediabetic patients believed that mHealth intervention would help them in self-management and almost all prediabetic patients exhibited their intention to use mHealth apps.

**Figure 2:**
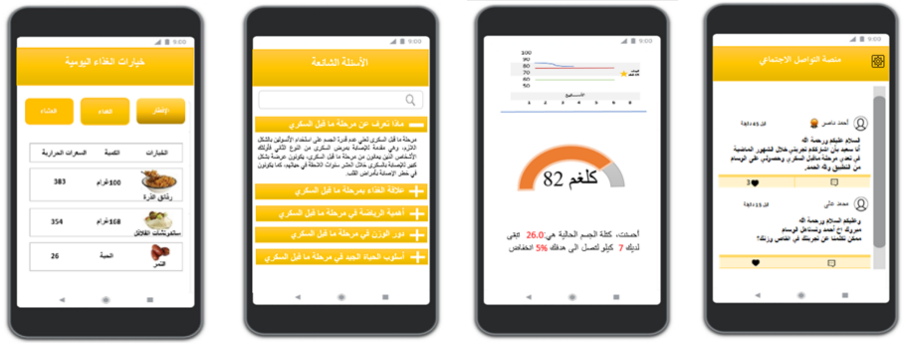
Sample of user interfaces in the proposed prototype.

From contextual inquiry and value specification phases, it seems that patients’ insights regarding the use, barriers to use, and preferred mHealth features are essential in understanding the role and usefulness of mobile health technology [57]. Therefore, we have elicited key intervention elements that were deemed important by both health care providers and prediabetic patients in KSUMC hospitals. Health care provider values and insights about the most impactful intervention strategies currently used in the practice have shaped the overall idea of the proposed design features of our mHealth app. Challenges and barriers as well as patient insights about their current use of well-being and health apps for the sake of their current health condition have helped us to define the key features of the proposed mHealth app. Given that the management of chronic diseases such as prediabetes has mostly relied on patient compliance to recommendations that occur outside the health care setting, the constant use of mHealth still represents a major challenge [58]. This study builds on Klansja and Pratt’s framework of 5 behavioral intervention strategies enabled by smartphones and the key components of lifestyle change programs in preventing type 2 diabetes [9,22]. Consequently, a low fidelity prototype was developed to present the proposed mHealth features for prediabetes self-management as illustrated in Figure 2.

The prototype phase showed that all patients who participated in this study indicated a significant need for the proposed features of the mHealth app regardless of their gender, age, and educational levels. In our study, we reviewed several mHealth apps recognized by the US Centers for Disease Control and Prevention for the sake of realizing the current status of diabetes prevention-related mHealth apps. We found that these apps were designed to encompass most of the features found in the well-being and health apps used by patients who participated in this study such as Fitbit, Samsung Health, iHealth, Apple Health, and VitaDock. Features like food, weight, and BMI tracking and blood glucose monitoring were very common. For example, Noom and Omada are well-known apps whose users have shown significant improvements in terms of weight loss which can be an excellent proxy for the risk of developing type 2 diabetes in the future [59,60]. These apps provide self-monitoring tools such as smart scales to manage weight and step count systems to track physical activity. However, these apps provide what may be considered the most important components of prediabetes intervention, personalized health coaching and group support, which are usually provided through text messaging [9]. A systematic review of all currently available diabetes apps for the operating systems iOS and Android indicated that more than one-half of well-being and health apps for DM provide one function, the language of the dominant app was English, and most respondents go beyond the paid mobile apps. Additionally, the number of functions in these apps was conversely correlated with usability [61]. Another study showed a significant improvement in monitoring glucose levels among adults with type 1 diabetes [62].

The prototype user interface proposed in this study showed that all 5 components of the lifestyle change program by Kirley and Sachdev, guided by Klansja and Pratt framework [9,22], are essential to make the mHealth app as usable as possible. Consequently, this study suggested that the constituent elements of a successful mHealth intervention for prediabetic patients should encompass a key features such as evidence-based contents of self-monitoring of diet, physical activity, and weight management features. Furthermore, the dietary options should consider alternatives in some cultural dishes with foods that are rich in carbohydrates that prediabetics should avoid. Additionally, a didactic curriculum of health education tailored to patient characteristics should be accompanied by physical activity and weight management features [63]. This can be delivered either by predefined notifications or text messaging as this was the most preferred means of coaching by both health care providers and prediabetic patients. To avoid patients opting out of the prediabetes management intervention and to ensure regular use of the proposed mHealth app, motivation elements should accompany the key features proposed. The motivation elements will help persuade patients to achieve recommended goals and constantly update their profile with achievements to allow more recommendations to be suggested. The proposed mHealth app should promote self-knowledge by facilitating access to important health information. This can be either in an interactive form such as quizzes that repeatedly pop up or static such as a list of frequently asked questions. Finally, the language of the contents must not be a barrier to the use of the mHealth intervention. Therefore, the proposed mHealth app should support the Arabic language in addition to English since the majority of Saudi Arabia’s citizens are Arabic native speakers.

### Limitations

Limitations of this study may include the cross-sectional design, sample size, time constraint, and confined group of patients. Furthermore, the study was done in a single round only for each phase. This study could be used as a baseline for future adoption of the app in the clinical practice context. Future work can then build on these findings and conduct as many iterative processes as needed to verify the findings. Future research also may consider qualitative methods such as interviews and focus groups approach to explore further insights about the current situation of the prediabetes management intervention. Another potential limitation is the low response rate of patients, which may not represent the whole population of prediabetic patients in KSUMC hospitals; this might be attributed to the short time of the study. Therefore, future work should overcome this issue in terms of encouraging prediabetic patients to participate in the study and allow for a sufficient period to conduct the study to ensure generalization.

### Conclusion

This study provided real-world insights into the development of mHealth apps for diabetes prevention by involving both health care providers and prediabetic patients in KSUMC hospitals. The development of the proposed mHealth app for prediabetes using the CeHRes guidelines provided a careful understanding of its content and design. Prediabetic patients who participated in this study exhibited their willingness to use the proposed mHealth app for self-management. Therefore, the proposed app, which comprises all necessary features, could contribute to a significant improvement of their self-management and changes in their lifestyle. The results of this study could be used as a baseline to further improve the adoption of the mHealth app for self-management of prediabetic patients.

## Appendix

Multimedia Appendix 1 Healthcare provider questionnaire.

Multimedia Appendix 2 Patient questionnaire (Arabic version).

Multimedia Appendix 3 Evaluation form of the proposed mHealth features.

Multimedia Appendix 4 Ethical approval letter.

## Abbreviations

CeHRes: Centre for eHealth Research
DM: diabetes mellitus
KSUMC: King Saud University Medical City
KKUH: King Khalid University Hospital
KAUH: King Abdulaziz University Hospital
mHealth: mobile health
