# Supplementary material for: Exploring the Constituent Elements of a Successful Mobile Health Intervention for Prediabetic Patients in King Saud University Medical City Hospitals in Saudi Arabia: Cross-sectional Study
Source: JMIR Form Res. 2021 Jul 20;5(7):e22968. doi: 10.2196/22968 (PMC8335605; doi:10.2196/22968)
Supplement: Multimedia Appendix 1 [file formative_v5i7e22968_app1.pdf]

This is 8 questions survey which is aimed to investigate the current practices in KSUMC for prediabetic patients.

The following survey is related to the research approval no.E-19-4118 with title: What should be the constituents elements of successful Mobile Health intervention for pre-diabetic patients in King Saud University Medical City's hospitals?

* Required

1. **Are you** *

   Mark only one oval.

   - Physician
   - Health educator
   - Dietitian

2. **Gender** *

   Mark only one oval.

   - Male
   - Female

3. **Age** *

   Mark only one oval.

   - 20-29
   - 30-39
   - 40-49
   - 50-59
   - 60 and more

4. **Which setting you are working at right now?** *

   Mark only one oval.

   - King Khalid University Hospital
   - King Abdullaziz University Hospital

5. **What is/are the most impactful intervention technique/s you used in your clinic for patients with prediabetes?** *

   Mark only one oval.

   - Medication
   - Medication + healthy diet plan
   - Medication + healthy diet plan + physical exercise plan
   - healthy diet plan + physical exercise plan
   - Other: ____________________

6. **What are the barriers or challenges facing the current interventions for prediabetic patients to prevent diabetes? For example, patient's adherence to instructions or loss to follow up with patient etc.** *

   ____
   ____
   ____
   ____
   ____

7. **Do you or any of your team members communicate with patients remotely? If so, by what means?** *

   Mark only one oval.

   - Phone call
   - Text messaging
   - Email
   - No communication outside the clinic working hours

8. **If there is a dedicated mobile application that use evidence based content for patient with prediabetes to help them with their medication/healthy diet/ physical activity plans?Would you recommend it to your patients?** *

   Mark only one oval.

   - Yes, I believe it can help them
   - Not sure if it can help them
   - No, I believe it will not help them

Powered by
Google Forms
