# Supplementary material for: Exploring the Constituent Elements of a Successful Mobile Health Intervention for Prediabetic Patients in King Saud University Medical City Hospitals in Saudi Arabia: Cross-sectional Study
Source: JMIR Form Res. 2021 Jul 20;5(7):e22968. doi: 10.2196/22968 (PMC8335605; doi:10.2196/22968)
Supplement: Multimedia Appendix 2 [file formative_v5i7e22968_app2.pdf]

استبيان في ثلاث دقائق عن دور التطبيقات الصحية في الهواتف الذكية في تجنب مرض السكري

هذه الاستبانة هي جزء من متطلب درجة الماجستير و الحاصلة على الموافقة رقم E-19-4118 من اخلاقيات البحوث العلمية بكلية الطب بجامعة الملك سعود.
ويعتبر إكمال هذه الاستبانة موافقة منكم على المشاركة في هذه الدراسة.
شكراً لكم على تعاونكم
للتواصل مع الباحث الرئيسي
فايز الشهري
afayz@ksu.edu.sa

* Required

1. **السؤال 1: اختر** *

Mark only one oval.

- ذكر
- أنثى

2. **السؤال 2: العمر** *

Mark only one oval.

- 20-24
- 25-29
- 30-34
- 35-39
- 40-44
- 45-49
- 50-54
- 55-59
- 60-65

3. **السؤال 3: المستوى التعليمي** *

Mark only one oval.

- اقراء واكتب (لايوجد مؤهل)
- ابتدائي
- متوسط
- ثانوي
- جامعي
- دراسات عليا (ماجستير او دكتوراه)

4. **السؤال 4:متى بدأت في المتابعة في العيادة لتجنب مرض السكري ؟** *

Mark only one oval.

- اقل من سنة
- سنة الى سنتين
- سنتين الى ثلاث سنوات
- ثلاث سنوات الى خمس سنوات
- اكثر من خمس سنوات

5. **السؤال 5 :ماهي الإجراءات الطبية المتبعة معك لتخطيء حالتك الصحية الحالية؟** *

Mark only one oval.

- ادوية لخفض السكر فقط
- ادوية لخفض السكر + برنامج غذائي (مثال:تعليمات في منشورات عن نوعية الأغذية الصحية)
- ادوية لخفض السكر + برنامج غذائي + برنامج لممارسة التمارين الرياضية بإنتظام (مثال:تعليمات في منشورات عن تمارين يومية لهدف انقاص وزنك)
- بدون ادوية فقط برنامج غذائي + برنامج لممارسة التمارين الرياضية بإنتظام

6. السؤال 6:ما هي الصعوبات التي تواجهك والتي من الممكن أن تعيقك عن الإستمرار والاستفادة من البرنامج العلاجي الحالي؟ (يمكنك اختيار اكثر من سبب) *

Check all that apply.

- [ ] اجد صعوبة في الالتزام في مواعيد أخذ الأدوية
- [ ] اجد صعوبة في الالتزام بالبرنامج الغذائي
- [ ] أجد صعوبة بالالتزام بممارسة الرياضة بانتظام
- [ ] اجد صعوبة في الالتزام بالحضور لمواعيدي بالمستشفى
- [ ] ليس لدي حافز نفسي يجعلني التزم بجميع ماسبق

7. السؤال 7:هل تستخدم احد التطبيقات الصحية على الهواتف الذكية للعناية بصحتك *

Mark only one oval.

- ( ) نعم
- ( ) أحياناً
- ( ) لا

8. اذا كان جوابك "نعم او احيانا" اذكر اسم التطبيق (اختياري)

____

9. السؤال 8:عندما تريد البحث عن معلومات تخص السكري، ما هي المصادر التي تلجأ اليها: *

Mark only one oval per row.

	دائماً	أحياناً	أبداً
ابحث في وسـائل التواصل الإجتماعي (مثل: تويتر، فيس بوك، واتساب، تليقرام)	( )	( )	( )
ابحث في مواقع الإنترنت بواسطة محرك البحث قوقل	( )	( )	( )
ابحث في اليوتيوب عن فيديو يخص استفساري	( )	( )	( )

10. السؤال 9:ما هي الصعوبات التي قد تواجهك عموماً عند استخدام أي تطبيق صحي على هاتفك الجوال؟ (تقييم الصعوبات 5= صعب جداً 0= ليس صعب) *

Mark only one oval per row.

	ليس صعب 0	1	2	3	4	5 صعب جداً
لغة التطبيق (مثال: التطبيق لا يدعم اللغة العربية	( )	( )	( )	( )	( )	( )
تعلّم كيفية استخدام التطبيق	( )	( )	( )	( )	( )	( )
فهم أهداف التطبيق	( )	( )	( )	( )	( )	( )
التنقل بين صفحات التطبيق/يستغرق وقت طويل للوصول للصفحة المطلوبة	( )	( )	( )	( )	( )	( )
حافز واستعداد داخلي لاستخدام هذه التطبيقات	( )	( )	( )	( )	( )	( )

11. السؤال 10:لو كان هناك تطبيق صحي متخصص يساعد في التجنب المبكر لمرض السكري، هل لديك استعداد لإستخدامة بإستمرار؟ *

Mark only one oval.

- ( ) نعم
- ( ) لا
- ( ) ربما

12. السؤال 11:في اي مستشفى تراجع؟ *

Mark only one oval.

- ( ) مستشفى الملك خالد الجامعي
- ( ) مستشفى الملك عبدالعزيز الجامعي

13. هل لديك اي تعليق اخر؟ (اختياري)

____
____
____
____
____

Powered by
