# Supplementary material for: Exploring the Constituent Elements of a Successful Mobile Health Intervention for Prediabetic Patients in King Saud University Medical City Hospitals in Saudi Arabia: Cross-sectional Study
Source: JMIR Form Res. 2021 Jul 20;5(7):e22968. doi: 10.2196/22968 (PMC8335605; doi:10.2196/22968)
Supplement: Multimedia Appendix 3 [file formative_v5i7e22968_app3.pdf]

فيما يلي مقترح لمحتويات تطبيق صحي متخصص في التجنب المبكر لمرض السكري، قيم المحتويات حسب احتياجك الشخصي.

هذه الاستبانة هي الجزء الثاني من الدراسة الحاصلة على الموافقة رقم E-19-4118 من اخلاقيات البحوث العلمية بكلية الطب بجامعة الملك سعود.
يعتبر اكمال هذه الاستبانة موافقة منكم على المشاركة في هذة الدراسة.
ملاحظة: صور الواجهات بالأسفل هي شكل تقريبي لاعطاء فكرة عن كيفية عمل التطبيق لمحتويات مختلفة لذا قد يكون بعضها مفيد والبعض الأخر غير مفيد حسب رأيكم (يفضل تدوير شاشة الهاتف الى الاتجاه الافقي)
قيم المحتوى حسب احتياجك من "احتاجه جداً" الى "لا احتاجه ابداً" يمكن تحريك الخيارات من يمين الى يسار.

* Required

1. **(المتابعة الذاتية) محتوى 1:يتيح التطبيق خاصية لعد الخطوات اليومية ذاتياً بالإضافة الى تذكيرك بالمسافة المتبقية لكي تنجز المسافة اليومية المطلوبة مع امكانية حصول المستخدم على نقاط و أوسمه عند تحقيق الهدف اليومي.** *

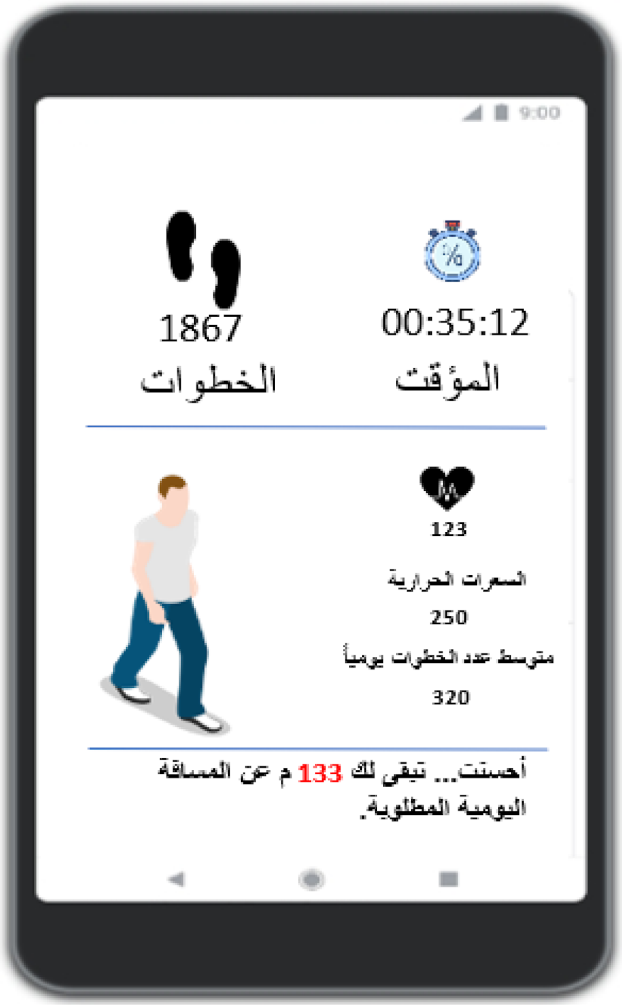

Mark only one oval per row.

	احتاجه جداً	احتاجه الى حد ما	محايد	لا احتاجه	لا احتاجه ابداً
عداد الخطوات الذاتي	⬭	⬭	⬭	⬭	⬭
الحصول على نقاط و أوسمه عند تحقيق الهدف اليومي من المشي	⬭	⬭	⬭	⬭	⬭

2. **(المتابعة الذاتية)محتوى 2: (للاشخاص الذين يستخدمون جهاز قياس السكرفقط) يتيح التطبيق حاسبة لحساب نسبة السكر في الدم التي تدخلها بنفسك من جهاز قياس السكر. ويتيح التطبيق عرض القراءات المدخلة خلال اسبوعين او 3 أشهر او 6 أشهر** *

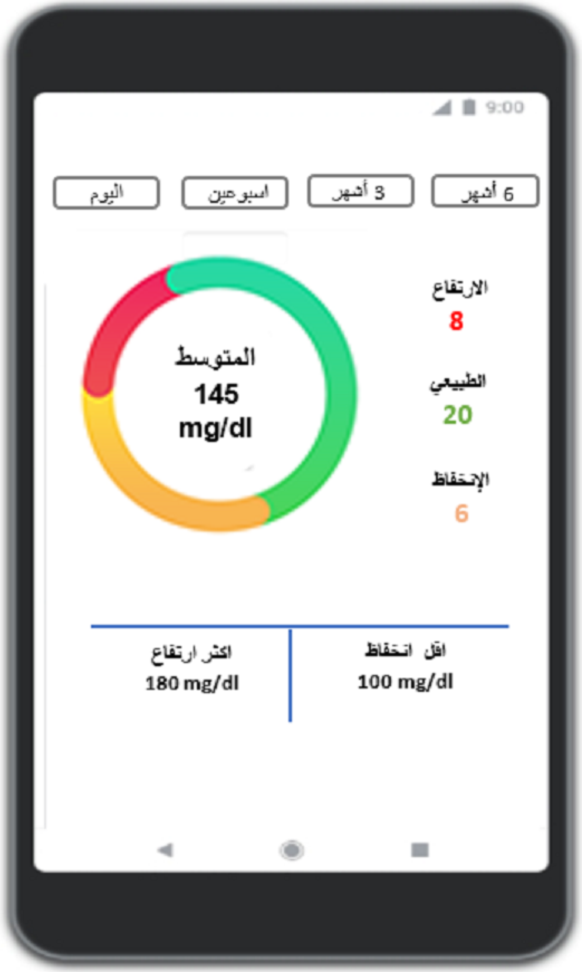

Mark only one oval per row.

	احتاجه جداً	احتاجه الى حد ما	محايد	لا احتاجه	لا احتاجه ابداً
حاسبة نسبة السكر	⬭	⬭	⬭	⬭	⬭

3. (المتابعة الذاتية)محتوى 3: يوفر التطبيق خيارات متنوعة للغذاء اليومي ويتيح معلومات عن كمية السعرات الحرارية فيها. *

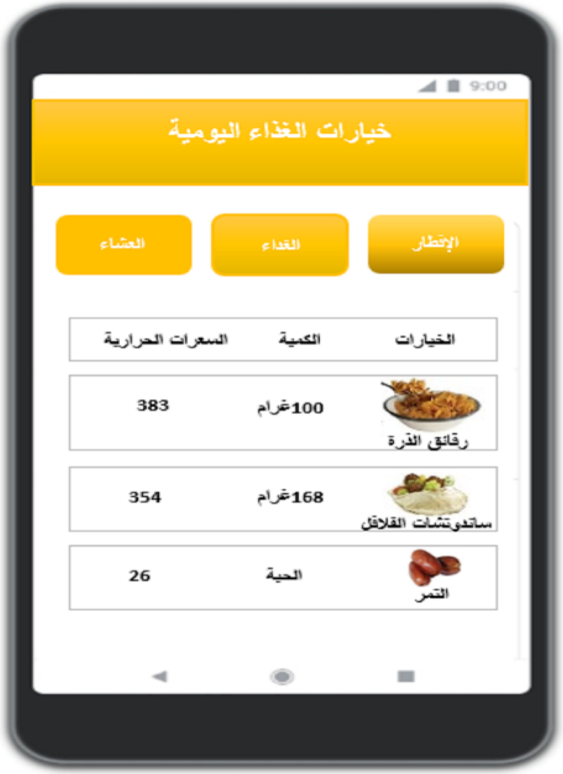

Mark only one oval per row.

	احتاجه جدا	احتاجه الى حد ما	محايد	لا احتاجه	لا احتاجه ابدا
خيارات الغذاء اليومي	◯	◯	◯	◯	◯

4. (المتابعة الذاتية)محتوى4: يتيح التطبيق اظهار التنبيهات المهمه التي يضبطها المستخدم مثال:مواعد اخذ الادوية/الوجبات/قراءات الدم/اواخذ قياس الوزن. *

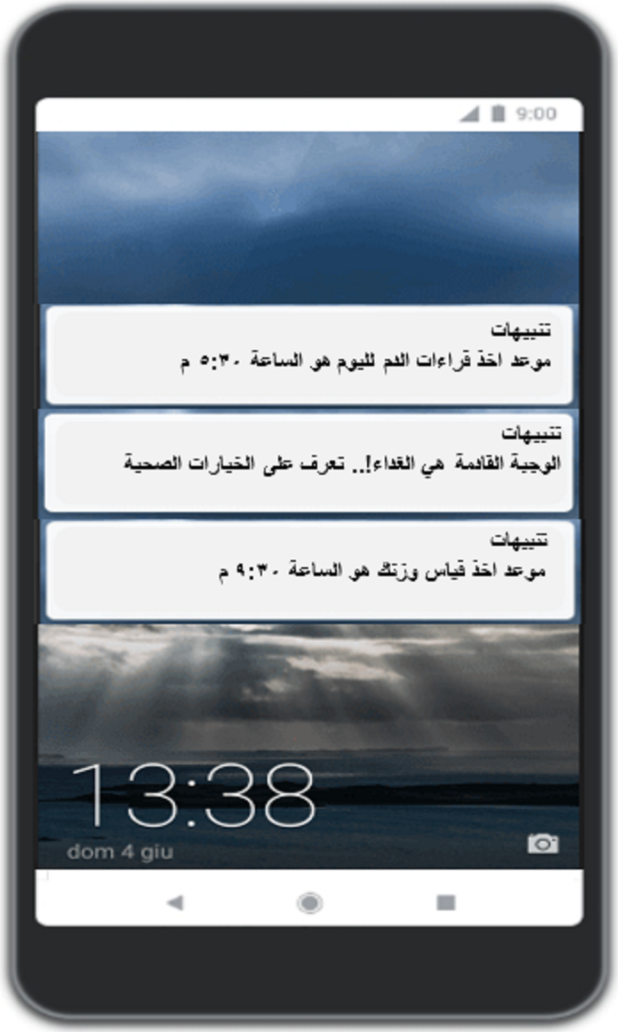

Mark only one oval per row.

	احتاجه جدا	احتاجه الى حد ما	محايد	لا احتاجه	لا احتاجه ابدا
التنبيهات و التذكير	◯	◯	◯	◯	◯

5. (الإشراف الطبي)محتوى 5: يتيح التطبيق التواصل للاستشارة عن طريق محادثة هاتفية مع طبيبك او اخصائي التغذية. *

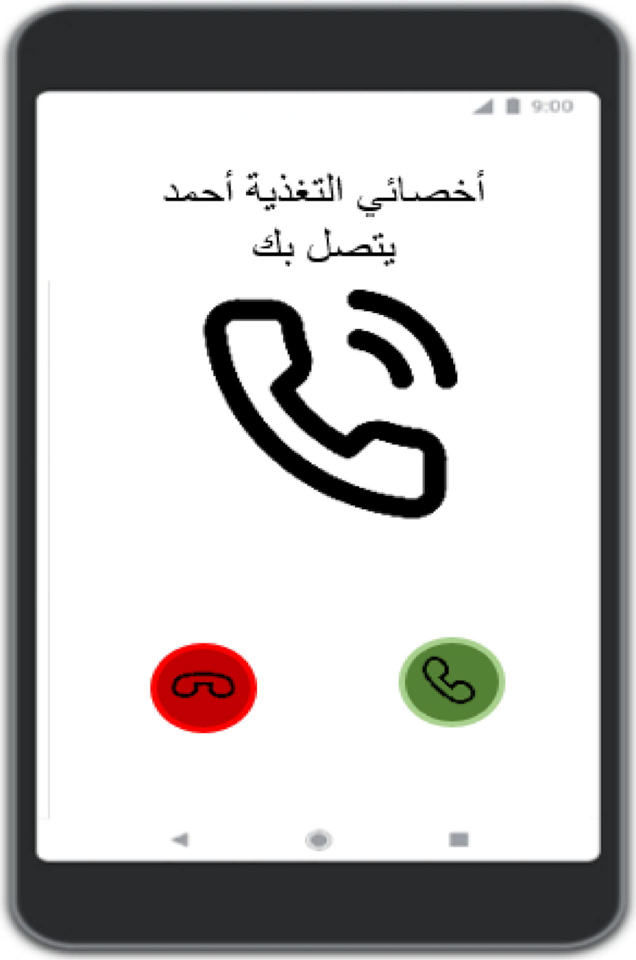

Mark only one oval per row.

	احتاجه جدا	احتاجه الى حد ما	محايد	لا احتاجه	لا احتاجه ابدا
المحادثة الهاتفية عبر التطبيق	◯	◯	◯	◯	◯

6. (الإشراف الطبي)محتوى 6: يتيح التطبيق التواصل للاستشارة عن طريق محادثة نصية مع طبيبك او اخصائي التغذية. *

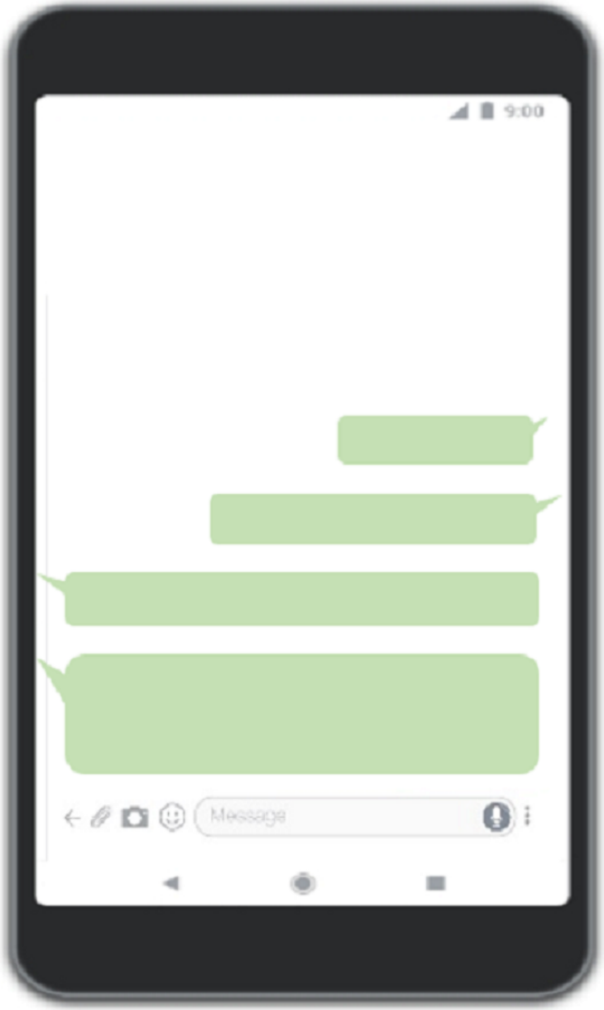

Mark only one oval per row.

	احتاجه جدا	احتاجه الى حد ما	محايد	لا احتاجه	لا احتاجه ابدا
المحادثات النصية	◯	◯	◯	◯	◯

7. (تطوير المهارات)محتوى 7: يتيح التطبيق اسئلة بشكل شبه يومي لتطوير مهارات العادات الصحية مع امكانية حصول المستخدم على نقاط و اوسمه عند الإجابة الصحيحة. *

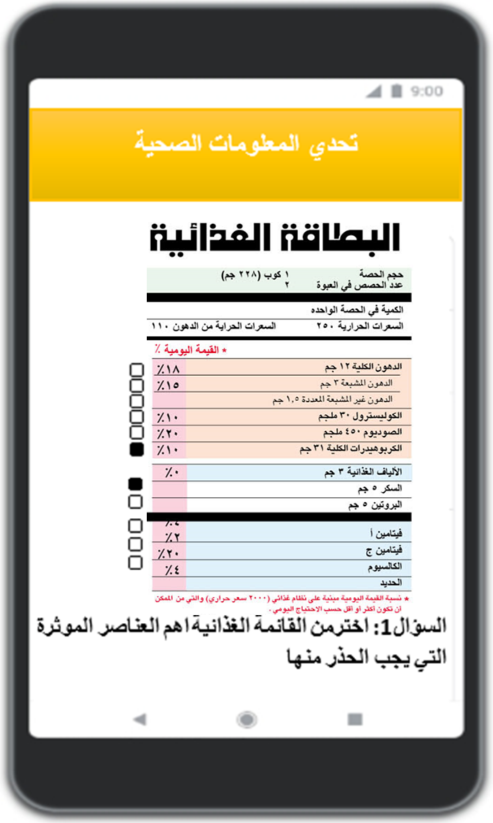

Mark only one oval per row.

	احتاجه جدا	احتاجه الى حد ما	محايد	لا احتاجه	لا احتاجه ابدا
اسئلة تطوير المهارات	⬭	⬭	⬭	⬭	⬭
الحصول على نقاط و اوسمه عند الاجابات الصحيحة	⬭	⬭	⬭	⬭	⬭

8. (المتابعة الذاتية)محتوى 8: يتيح التطبيق متابعة الوزن وحساب كتلة الجسم في كل مره يدخل المستخدم فيها وزنه بشكل اسبوعي مع امكانية حصول المستخدم على نقاط و اوسمه عند تحقيق الهدف الاسبوعي في انخفاض في الوزن. *

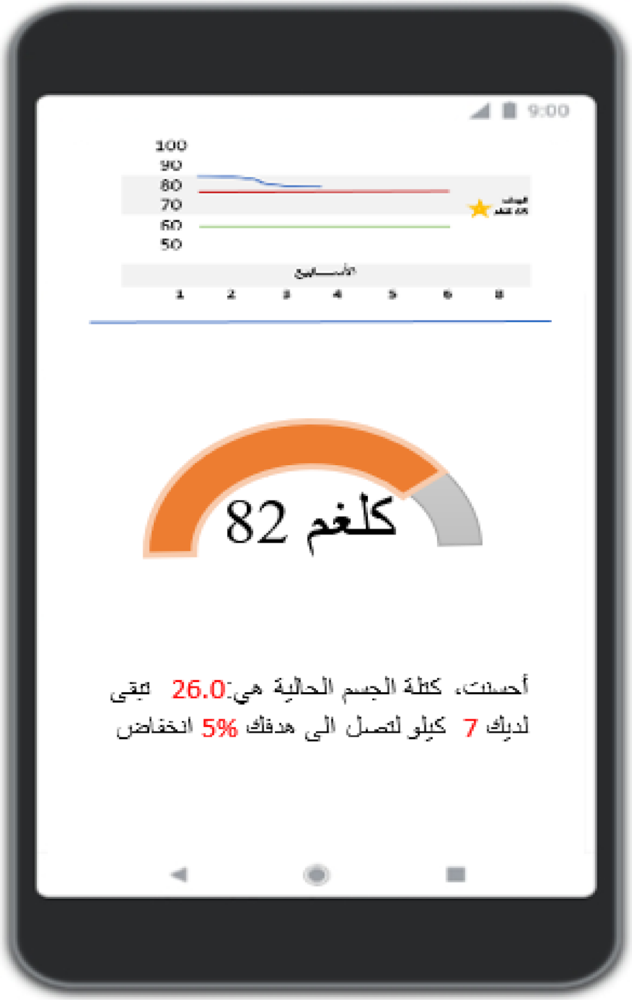

Mark only one oval per row.

	احتاجه جدا	احتاجه الى حد ما	محايد	لا احتاجه	لا احتاجه ابدا
حاسبة الوزن وكتلة الجسم	⬭	⬭	⬭	⬭	⬭
الحصول على نقاط و اوسمه عند تحقيق الهدف الاسبوعي	⬭	⬭	⬭	⬭	⬭

9. (تواصل اجتماعي) محتوى 9:يتيح التطبيق خاصية محادثات في (مجموعة أو خاصة) تتيح لك النقاش مع اصدقاء يشاركونك نفس الحالة الصحية يتشاركون النصائح من خلال تجاربهم ووصولهم بنجاح لاهدافهم في التحكم في حالتهم الصحية. *

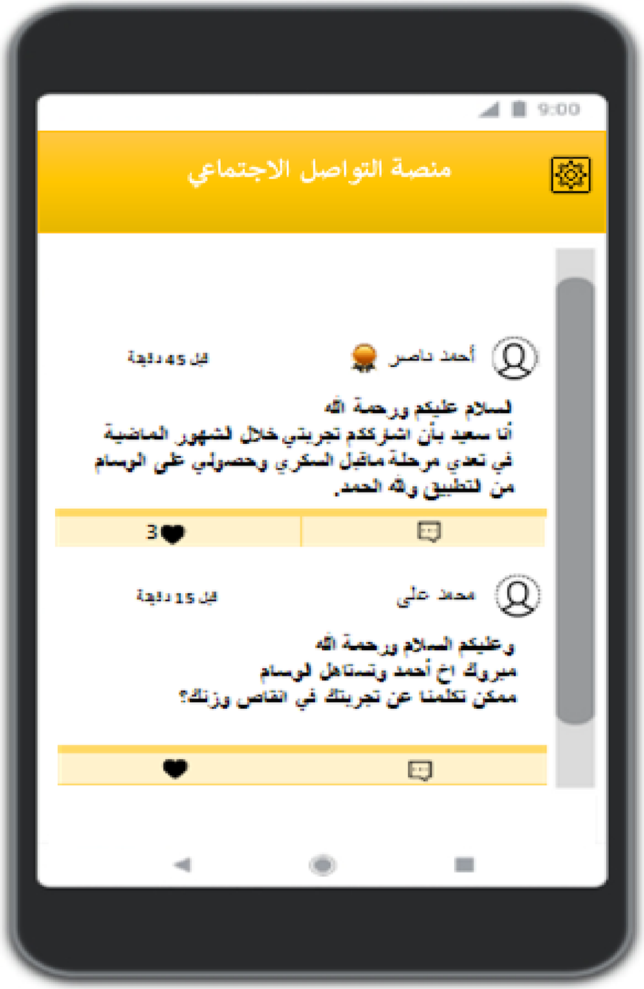

Mark only one oval per row.

	احتاجه جدا	احتاجه الى حد ما	محايد	لا احتاجه	لا احتاجه ابدا
محادثات في مجموعة	⬭	⬭	⬭	⬭	⬭
محادثات خاصة (واحد لواحد)	⬭	⬭	⬭	⬭	⬭

10. (تطوير المهارات)محتوى 10 : يوفر التطبيق خاصية الاسئلة الشائعة والتي تحتوي على معلومات اساسية عن حالة ماقبل السكري وكيفية تجنب خطر الاصابة بالسكري. *

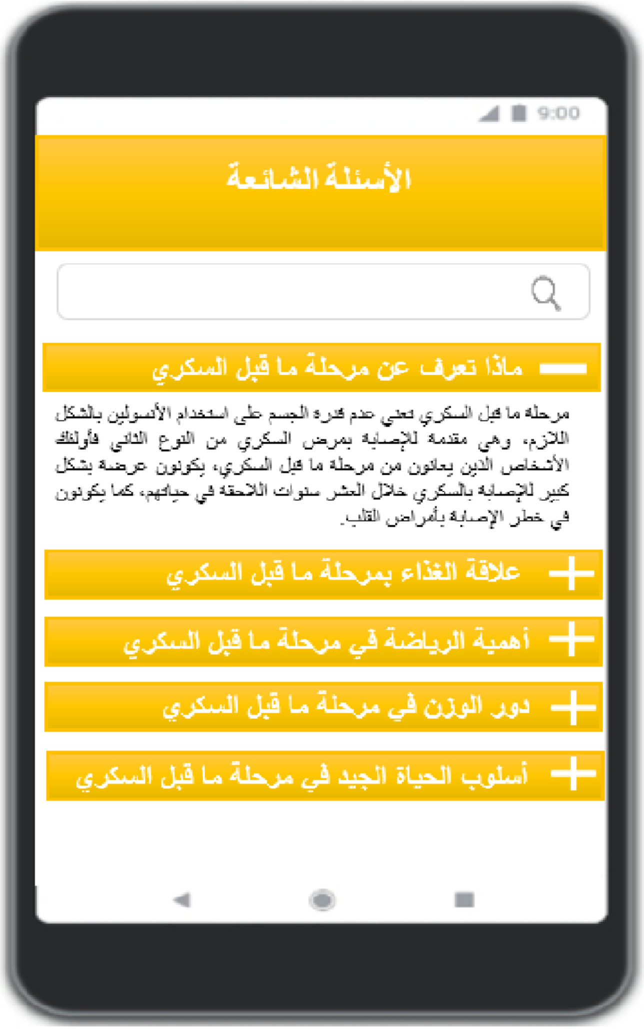

Mark only one oval per row.

	احتاجه جدا	احتاجه الى حد ما	محايد	لا احتاجه	لا احتاجه ابدا
الأسئلة الشائعة	⬭	⬭	⬭	⬭	⬭

11. سؤال 11:اختار النوع *

Mark only one oval.

- ⬭ ذكر
- ⬭ أنثى

12. سؤال 12:العمر *

____

13. السؤال13 :متى بدأت في المتابعة في العيادة لتجنب مرض السكري ؟ *

Mark only one oval.

- اقل من سنة
- سنة الى سنتين
- سنتين الى ثلاث سنوات
- ثلاث سنوات الى خمس سنوات
- اكثر من خمس سنوات

14. السؤال14 :ماهي الإجراءات الطبية المتبعة معك حاليا؟ *

Mark only one oval.

- ادوية لخفض السكر فقط
- ادوية لخفض السكر + برنامج غذائي (مثال:تعليمات في منشورات عن نوعية الأغذية الصحية)
- ادوية لخفض السكر + برنامج غذائي + برنامج لممارسة التمارين الرياضية بإنتظام (مثال:تعليمات في منشورات عن تمارين يومية لهدف انقاص وزنك)
- بدون ادوية فقط برنامج غذائي + برنامج لممارسة التمارين الرياضية بإنتظام

15. السؤال 15: في اي مستشفى تراجع؟ *

Mark only one oval.

- مستشفى الملك خالد الجامعي
- مستشفى الملك عبدالعزيز الجامعي

16. هل لديك اقتراحات (اختياري)

Powered by
Google Forms
