# Supplementary material for: Exploring the Constituent Elements of a Successful Mobile Health Intervention for Prediabetic Patients in King Saud University Medical City Hospitals in Saudi Arabia: Cross-sectional Study
Source: JMIR Form Res. 2021 Jul 20;5(7):e22968. doi: 10.2196/22968 (PMC8335605; doi:10.2196/22968)
Supplement: Multimedia Appendix 4 [file formative_v5i7e22968_app4.pdf]

Kingdom of Saudi Arabia
King Saudi University (034)
p.o. Box 7805 Riyadh 11472
Tel: +966 11 467 00 11
Fax: +96611 467 19 92
http://medicalcity.ksu.edu.sa

المملكة العربية السعودية
جامعة الملك سعود (٠٣٤)
ص.ب ٧٨٠٥ الرياض ١١٤٧٢
هاتف: ٩٦٦١١٤٦٧٠٠١١+
فاكس: ٩٦٦١١٤٦٧١٩٩٢+

جامعة الملك سعود
King Saud University
1957

المدينة الطبية الجامعية

12.11.19 (15.03.1441)
Ref. No. 19/0112/IRB

To: **Mr. Fayz Osman Alshehri**
Department of Medical Education - Medical Informatics and ELearning Unit
King Saud University College of Medicine
King Saud University Medical City
Email: afayz@ksu.edu.sa
Principal Investigator

Subject: **Approval of Amendment of Research Project No. E-19-4118**

Study Title: "What should be the Constituent's Elements of Successful Mobile Health Intervention for Pre-Diabetic Patients in King Saud University Medical City?"

Type of Review: Expedite

Dear Mr. Fayz Osman Alshehri,

Reference to your letter dated 07-November-2019 requesting for approval of Modifications done in the above-mentioned research project which was reviewed and approved by the Institutional Review Board (IRB) in 05 August 2019 (04 Dhul Al-Hijjah 1440). Please be informed that the IRB has granted your request and approved the following changes:

1. Change of Study Title: From "What should be the Constituent's Elements of Successful Mobile Health Intervention for Pre-Diabetic Patients in King Khalid University Hospital?" To: What should be the Constituent's Elements of Successful Mobile Health Intervention for Pre-Diabetic Patients in King Saud University Medical City?
2. Addition of study population sample size: Healthcare Provider= 30, Prediabetes Patients = 281
3. Some changes in the survey questions for more clarifications (adding pictures of mobile app Interfaces)

You may continue with the conduct of this study using the above-mentioned approved modification. The IRB mandates regular submission of the study progress report every six months by the primary investigator. Otherwise, project approval will be suspended.

Thank you!

Sincerely yours,

Prof. Abdulrahman Alsultan
Chairman, Institutional Review Board
King Saud University College of Medicine
King Saud University Medical City
P.O. Box 7805 Riyadh 11472 K.S.A.
E-mail: aalsultan1@ksu.edu.sa

كلية الطب
لجنة أخلاقيات البحوث الطبية
College Medicine
Institutional Review Board (IRB)

/braezell

الرقم
التاريخ
المرفقات
